# Drug development studies supporting zileuton as a parenteral adjuvant to attenuate antibiotic-associated nephrotoxicity

**DOI:** 10.1128/aac.00287-25

**Published:** 2025-07-08

**Authors:** Cole S. Hudson, James E. Smith, Linh T. Vuong, Nicholas S. Teran, Nazanin Pouya, David Sheikh-Hamad, Xinli Liu, Shama Kajiji, Vincent H. Tam

**Affiliations:** 1 Department of Pharmacological & Pharmaceutical Sciences, University of Houston College of Pharmacy 15507, Houston, Texas, USA; 2 Department of Pharmacy Practice & Translational Research, University of Houston College of Pharmacy 15507, Houston, Texas, USA; 3 Division of Nephrology and Selzman Institute for Kidney Health, Department of Medicine, Baylor College of Medicine 171841, Houston, Texas, USA; 4 Center for Translational Research on Inflammatory Diseases (CTRID), Michael E DeBakey VA Medical Center 20116, Houston, Texas, USA; 5 Emergent System Analytics, LLC, Clinton, Connecticut, USA; University of Pittsburgh School of Medicine, Pittsburgh, Pennsylvania, USA

**Keywords:** antimicrobial resistance, nephroprotectant, drug formulation

## Abstract

Glycopeptide, polymyxin, and aminoglycoside antibiotics are among the most commonly used agents to treat drug-resistant bacterial infections; however, their clinical use is hindered by nephrotoxicity. We previously reported that zileuton has the potential to attenuate antibiotic-associated nephrotoxicity in an animal model. Here, we further report the development of a parenteral formulation and exploration of dosing strategies for zileuton. The solubility, stability, and multi-dose safety of two zileuton formulations were evaluated. Zileuton serum and renal tissue pharmacokinetics were evaluated after a single dose and compared to steady state after 10 days. Different dosing strategies of zileuton to attenuate vancomycin-, polymyxin B-, and amikacin-associated nephrotoxicity were evaluated in rats over 10 days. Two formulations (1 and 10 mg/mL) showed good multi-dose safety, with no significant changes in serum creatinine, alanine transaminase, or body weight observed following doses of 12 mg/kg daily for 10 days. Zileuton was well-distributed into renal tissue, and serum exposure was comparable to humans after a typical dose (600–2,400 mg). No drug accumulation at steady state was observed. Zileuton was found to reduce nephrotoxicity associated with vancomycin, polymyxin B, and amikacin in a dose-dependent manner. With the same daily dose, dose fractionation resulted in similar renal protection compared to once-daily dosing. These preliminary studies support that zileuton has the potential to be used as a parenteral adjuvant to attenuate antibiotic-associated nephrotoxicity. This would allow the optimal use of glycopeptides, polymyxins, and aminoglycosides to treat difficult infections. Further studies are warranted to repurpose zileuton as a nephroprotectant.

## INTRODUCTION

The global threat of antimicrobial resistance (AMR) is rapidly growing, increasing the burden of bacterial infections on human health and healthcare systems. In 2024, the CDC reported that infections caused by the seven most threatening hospital-onset pathogens, including carbapenem-resistant Enterobacterales and methicillin-resistant *Staphylococcus aureus* (MRSA), had increased by a combined 20% since 2019 (1). The dearth of safe, effective antibiotics for treating infections caused by these pathogens is growing, and new drug development by the pharmaceutical industry is not likely to provide viable options in time. Therefore, finding novel approaches to reduce the health and economic burden of infections caused by multidrug-resistant (MDR) bacteria is an urgent priority.

Resistance to first-line antibiotics, such as carbapenems, is widespread and has rendered them ineffective treatment options. For example, a retrospective study of gram-negative bacteria collected from hospitals from 2021 to 2022 found that carbapenem resistance rates among *Acinetobacter baumannii*, *Klebsiella pneumoniae*, and *Pseudomonas aeruginosa* were 93%, 59%, and 42%, respectively (2). Glycopeptides, aminoglycosides, and polymyxins are three classes of antibiotics that have generally maintained *in vitro* activity against drug-resistant bacteria. A recent retrospective study of almost 1,000 clinical bacterial isolates found that 98.8% of gram-positive bacteria were susceptible to vancomycin (a glycopeptide), and 93.7% of gram-negative bacteria were susceptible to amikacin (an aminoglycoside) (3). Unfortunately, the clinical usage of these antibiotics is hindered by dose-limiting nephrotoxicity. For example, a study of 94 patients receiving vancomycin for MRSA infections found that 43% developed renal toxicity during the course of their treatment (4). Aminoglycosides and polymyxins are associated with renal toxicity rates of 58% and 60%, respectively (5, 6). Attenuation of nephrotoxicity would allow the optimal clinical use of these antibiotics to treat difficult infections and provide timely solutions to the AMR crisis.

Zileuton is an FDA-approved anti-inflammatory drug used for the treatment of chronic asthma by inhibiting the synthesis of pro-inflammatory molecules known as leukotrienes. Zileuton has been demonstrated by other investigators to have renal protective effects against various nephrotoxins (such as cisplatin) in preclinical models (7–9). We previously reported a pilot study demonstrating that zileuton had the potential to reduce kidney injury in an animal model (10). However, zileuton is water-insoluble (0.14 mg/mL) and is classified as biopharmaceutical classification system class II drug (11, 12). Zileuton is only available as tablets for oral administration, which is not reliable for the critically ill patient population who would benefit from its renal protective effects. Therefore, in this study, we developed a parenteral formulation of zileuton and explored optimal dosing strategies to attenuate glycopeptide-, aminoglycoside-, and polymyxin-associated kidney injury in a rat model.

## MATERIALS AND METHODS

### Chemicals and reagents

Zileuton powder (United States Pharmacopeia [USP]) was obtained from Supelco (Bellefonte, PA, USA). Piccolo comprehensive metabolic panels were purchased from Abaxis (Union City, CA, USA). Vancomycin hydrochloride (USP) was obtained from Slate Run Pharmaceuticals (Columbus, OH, USA). Amikacin sulfate (USP) was obtained from Sagent Pharmaceuticals (Schaumburg, IL, USA). Polymyxin B (USP) was purchased from AuroMedics Pharma (East Windsor, NJ, USA). Polyethylene glycol 400 (PEG-400) was purchased from Spectrum Chemical (New Brunswick, NJ, USA). Liquid chromatography-mass spectrometry (LC/MS)-grade water and acetonitrile were obtained from Supelco (Bellefonte, PA, USA).

### Zileuton assays and preparation of samples for quantification

A high-performance liquid chromatography (HPLC) method was developed and validated for *in vitro* stability studies, since the formulation composition was not preferred for liquid chromatography tandem mass spectrometry (LC-MS/MS) analysis. No interference of the formulation excipients was observed compared to pure drug solution. Briefly, formulation samples were diluted 100× or 1,000× in HPLC-grade water with 15 mg/L phenacetin as internal standard. Absorption at 230 nm was used for quantification. Mobile phase A was water +0.1% formic acid, and mobile phase B was acetonitrile +0.1% formic acid. A Kinetex EVO C18 column (100 × 2.1 mm internal diameter, 5 µm) with column oven temperature of 30°C and injection volume of 10 µL was used. Chromatographic separation was achieved with a flow rate of 0.3 mL/min and constant 30% mobile phase B. The linear range of the assay was 0.125–15 mg/L (the lower limit of quantification [LLOQ] = 0.0625 mg/L). The r^2^ of all calibration curves was >0.98. The intraday precision and accuracy for high, middle, and low concentration quality control standards were determined using six replicates of each concentration measured on the same day. The interday precision and accuracy were determined across three separate days. Intra- and inter-day precision and accuracy were acceptable (CV% and %error <10%).

In contrast, a previously reported LC-MS/MS method used for quantification of zileuton in serum was modified for renal tissue (10). Serum samples were prepared as previously described (10). A modified method was used for the preparation of kidney homogenate samples. Briefly, working solutions of zileuton in 2 × concentration dilutions (0.625–160 mg/L) were prepared in 40% methanol from a 10 mg/mL stock solution stored at −20°C. The internal standard working solution (20 mg/L) was prepared in methanol from a stock solution stored at −80°C. 25 µL of kidney homogenate was combined with 5 µL of zileuton working solution and 10 µL of internal standard solution. Cold acetonitrile (0.5 mL) was added, the mixture was vortexed for 0.5 minutes, and then centrifuged at 15,000 × *g* for 15 minutes. The supernatant was recovered, dried under a stream of air, and stored at −80°C until analysis. Standards and samples were reconstituted in 1 mL of water and centrifuged again at 15,000 × *g* for 15 minutes. The linear range of the assay was 0.125–32 mg/L. The LLOQ was 0.0625 mg/L. Each calibration curve was calculated using a 1/x weighting of the linear regression. Intra- and inter-day precision and accuracy were acceptable (CV% and error% < 10%). The extraction recovery was good (>80%) and the matrix effect was not significant (< ±10%).

### Solubility and stability studies

Zileuton equilibrium solubility was determined by shaking solvents with excess zileuton for 24 hours at 25°C followed by centrifugation at 10,000 × *g* for 10 minutes. The supernatant was collected, diluted in methanol, and the zileuton concentration was measured using UV spectroscopy (absorbance at 230 nm). Zileuton stability in ethanol (10 mg/mL) and two proprietary formulations (1 and 10 mg/mL, US patent application: PCT/US2023/072771) was determined using the HPLC method. Briefly, zileuton was dissolved and stored at various temperatures (−80, 4, 22, 37°C) for select time periods (1 day, 1 week, 1 month). At each time point, the concentration was measured in triplicate by dilution in water + IS and quantification by HPLC.

### Animals

Sprague Dawley male (325–350 g) and female (225–250 g) rats were purchased from Envigo (Indianapolis, IN, USA). All protocols were approved by the Institutional Animal Care and Use Committee of the University of Houston.

### Multi-dose safety experiments

To evaluate the safety of multiple doses of the zileuton formulations, rats were administered 12 mg/kg zileuton as a single daily dose for 10 days (*N* = 6 for 1 mg/mL formulation and *N* = 3 for 10 mg/mL formulation). Blood samples (0.2 mL) were collected from the tail tip and assayed for alanine transaminase (ALT) and serum creatinine levels (sCr) to assess hepatotoxicity and nephrotoxicity, respectively. Body weight was also measured longitudinally on selected days.

### Hemolysis assay

The hemolytic potential of the zileuton formulations was evaluated using a previously reported method with slight modification (13). Briefly, 0.45 mL of whole rat blood containing K2 EDTA (Innovative Research, IGRTSDWBK2E) was combined with 0.05 mL of the formulations, 10% Triton X-100 (positive control), or saline (negative control) and incubated at 37°C for 1 hour. Percent hemolysis was then determined spectrophotometrically as previously described (13).

### Zileuton pharmacokinetic studies

Three male and three female rats were administered zileuton (a single dose of 4 or 12 mg/kg) intraperitoneally, and serial blood samples (90 µL each) were taken over 12 hours from the tail tip. The blood samples were allowed to clot at room temperature and centrifuged at 10,000 × *g* for 10 minutes. The serum was then collected and stored at −20°C until analysis. Following quantification, serum concentrations at various time points were averaged, and the mean concentration was used for analysis.

To evaluate renal tissue pharmacokinetics, selected animals were euthanized at various time points. Both kidneys were then collected, weighed, homogenized in 5 mL of water, and stored at −20°C until analysis. Renal tissue concentrations were averaged and co-modeled with serum concentrations using a three-compartment model in ADAPT5 software. Serum area under curve (AUC) was compared to human AUC observed following a typical dose (19.2 mg*h/L for a 600 mg dose) and correlated to the human equivalent dose (HED), assuming dose linearity (14).

To evaluate dose accumulation, three male and three female rats were administered zileuton (12 mg/kg/day) intraperitoneally for 10 days. On the 10th day, serial blood samples were collected as described above. The serum concentrations were then averaged and compared to the serum concentration profile achieved with a single dose.

### Animal model of nephrotoxicity

Experimental details of the animal model using amikacin and polymyxin B were previously described (10). Similarly, animals were administered vancomycin (200 mg/kg) intraperitoneally once daily over 10 days. To explore the optimal zileuton dosing strategy, both dose escalation and fractionation designs were undertaken. Nephrotoxicity endpoint was defined as doubling of sCr concentration from baseline, which is equivalent to the “injury” category using the Risk, Injury, Failure, Loss, and End-stage kidney disease (RIFLE) criteria and could be thought of as moderate extent kidney injury. The onset of nephrotoxicity in different treatment cohorts was compared using a time-to-event (e.g., Kaplan-Meier) analysis and log-rank test. *P* values ≤ 0.05 were considered significant.

## RESULTS

### Zileuton solubility and stability

Zileuton equilibrium solubility in the 1 and 10 mg/mL formulations was determined to be 1.68 and 12.9 mg/mL, respectively, and solubility in ethanol was approximately 30 mg/mL.

The long-term stability (≥90% remaining) of zileuton stored at −20°C as a 10 mg/mL solution in ethanol was found to be >12 months. The stability of zileuton following dilution with the cosolvent system was determined at −80, 4, 22, 37°C (Table 1). At room temperature (22°C), zileuton was stable in the 1 and 10 mg/mL formulations for 16 hours and 4 weeks, respectively. Additionally, no physical instability (e.g., precipitation) was observed during storage at any temperature.

**TABLE 1:** Zileuton stability in the cosolvent formulation^*a*^

Temperature (°C)	1 mg/mL formulation	10 mg/mL formulation (days)
37	>8 hours	>3
22	>16 hours	>28
4	>1 day	>28
−80	>14 days	>28

^*a*^ Stability = ≥95% remaining.

### Multi-dose safety of zileuton in the parenteral formulation

**Fig 1:**
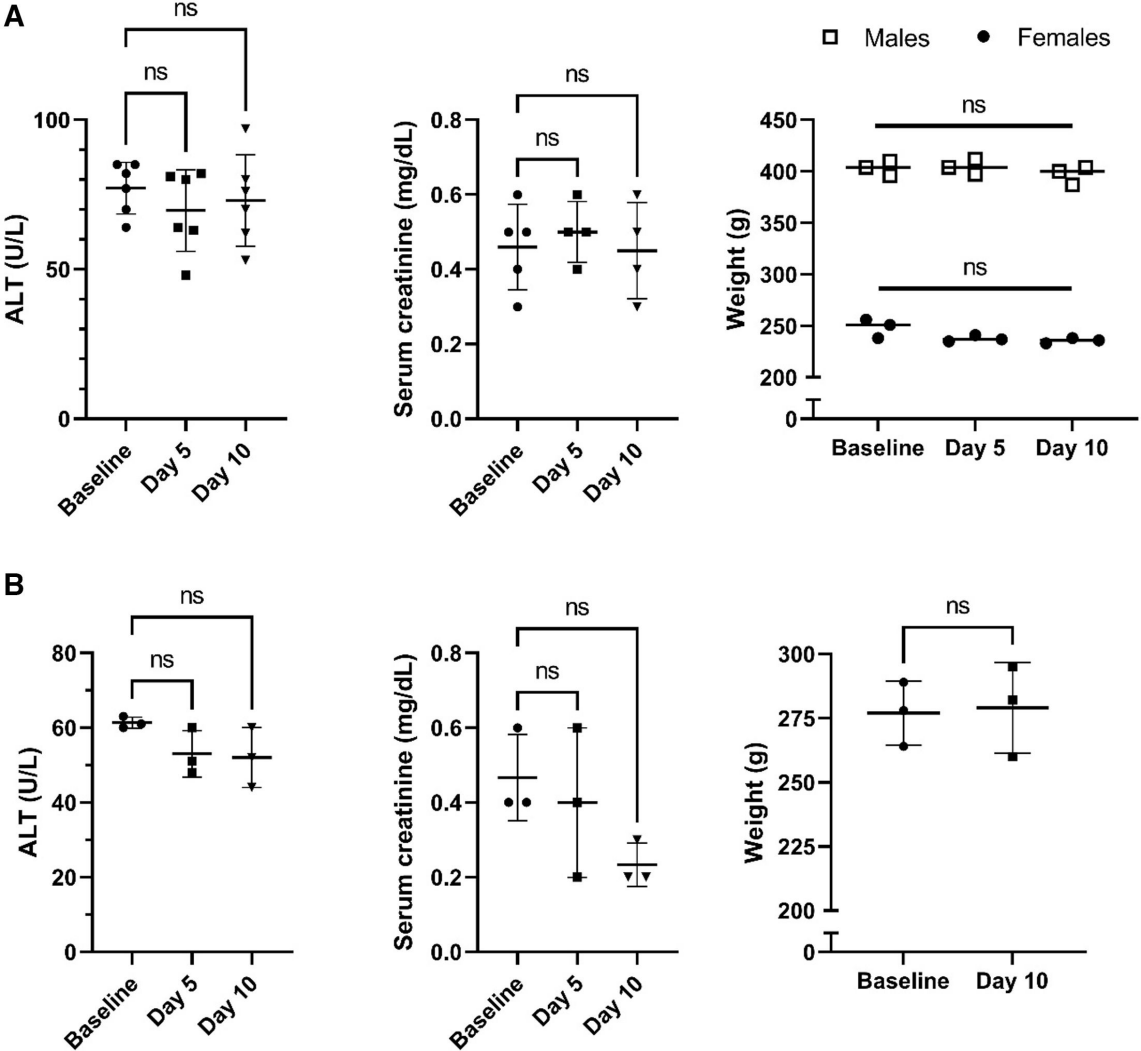
Multi-dose safety of zileuton (12 mg/kg) administered intraperitoneally in rats with the 1 mg/mL formulation (**A**) and 10 mg/mL formulation (**B**) for 10 days. Data are shown as mean ± SD. *N* = 6 (3 males and 3 females) for 1 mg/mL and *N* = 3 (females) for 10 mg/mL. ALT, alanine transaminase.

Compared to baseline, there were no significant changes in serum ALT (hepatocellular toxicity marker), sCr (renal function marker), or body weight observed over 10 days (Fig. 1). Additionally, there was no gross irritation of the injection site observed.

### Hemolytic potential

The 1 and 10 mg/mL zileuton formulations were found to result in 2% and 16% hemolysis, respectively. Generally, hemolysis values of <10% are considered non-hemolytic, while those exceeding 25% are considered hemolytic (13).

### Zileuton pharmacokinetics

**Fig 2:**
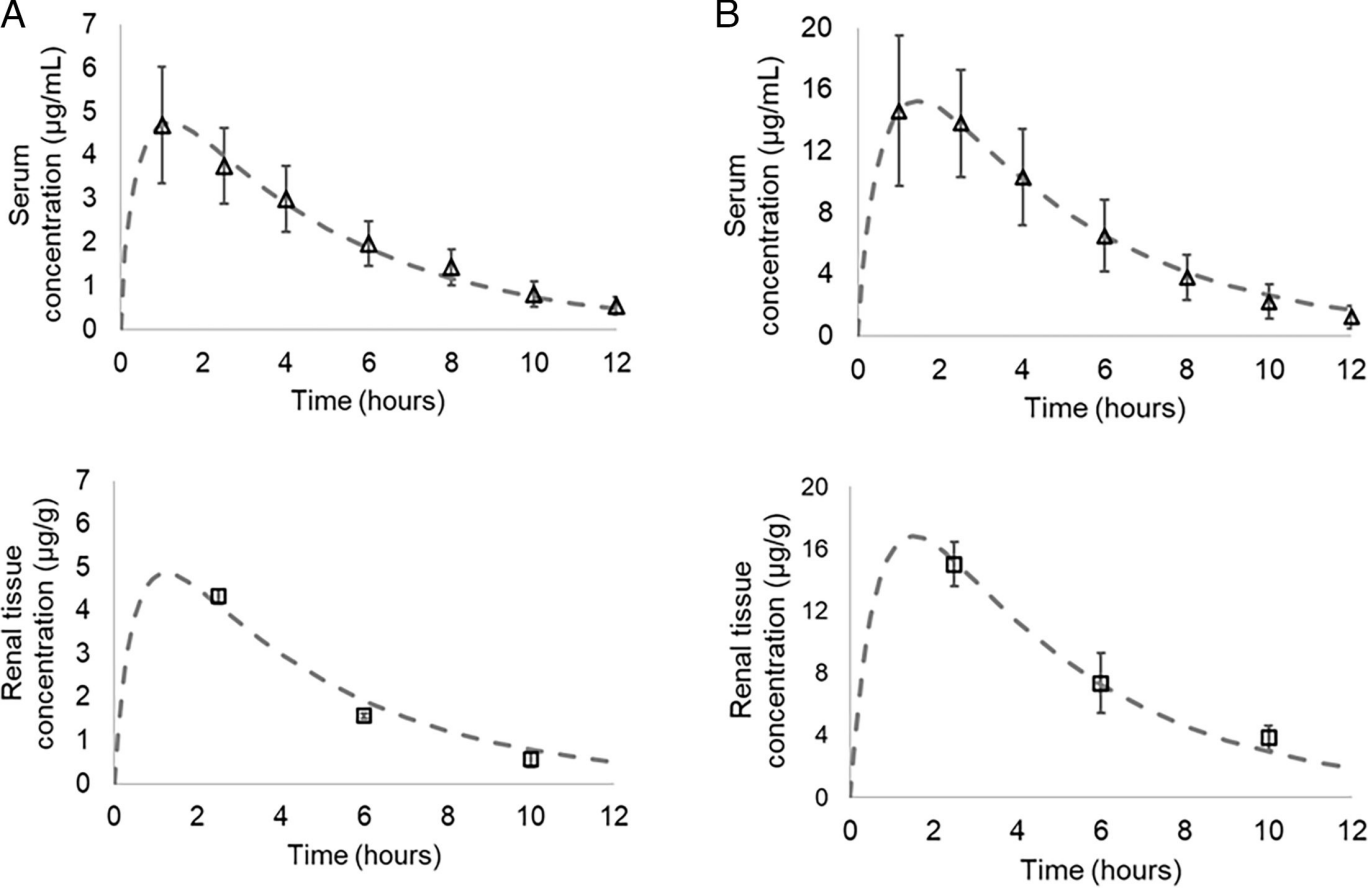
Zileuton pharmacokinetics in serum and renal tissue in rats after intraperitoneal administration of 1 mg/mL zileuton formulation. (**A**) 4 mg/kg and (**B**) 12 mg/kg. Data are shown as mean ± SD. Best-fit line is dashed. r^2^ >0.99 for all profiles. *N* = 6 for serum data points and *N* = 2 for renal tissue data points. A three-compartment model was fit to the serum and tissue data.

Zileuton serum and renal tissue concentrations were measured up to 12 hours following 4 and 12 mg/kg single doses with the 1 mg/mL formulation. Serum and tissue concentrations were reasonably well characterized (r^2^ ≥0.99 for all profiles) (Fig. 2), and the best-fit pharmacokinetic parameters were determined (Table 2). The AUC of renal tissue was similar to the serum AUC at both doses. AUC and maximum concentration (Cmax) increased approximately proportionally between the two doses. Tmax was 1 and 1.5 hours for 4 and 12 mg/kg doses, respectively.

**TABLE 2:** Zileuton serum/renal tissue pharmacokinetic parameters determined following intraperitoneal administration of the 1 mg/mL formulation in rats*^a^*^,*b*^

Dose	4 mg/kg	12 mg/kg
Vc (L/kg)	0.216	0.233
CL (L/hr*kg)	0.141	0.126
V_tissue_ (L/kg)	0.06	0.067
K_a_ (h^−1^)	1.81	1.41
Serum AUC (mg*h/L)	28.3	95.6
Tissue AUC (mg*h/L)	29.02	105.1
HED (mg)	880	3,000

^*a*^ Pharmacokinetic parameters were determined by co-modeling serum and renal tissue concentrations with a three-compartment model in ADAPT5.

^*b*^ Vc, central compartment; CL, clearance; V_tissue_, tissue compartment; Ka, absorption rate constant; AUC, area under the concentration-time curve; HED, human equivalent dose.

**Fig 3:**
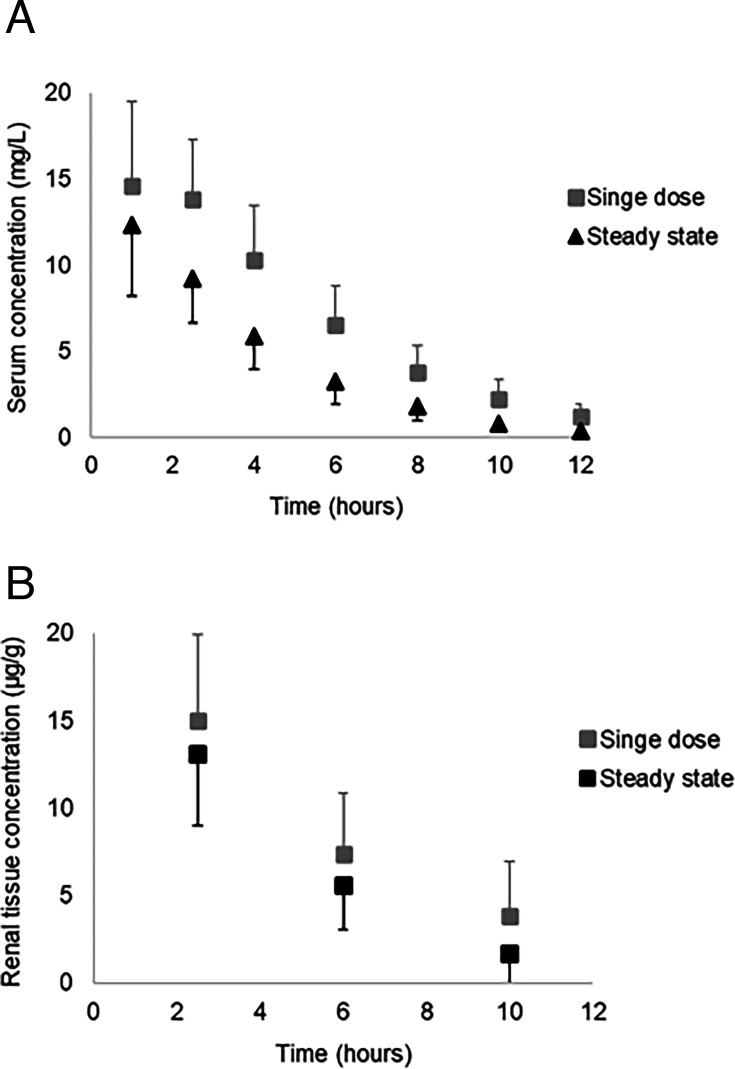
Accumulation of zileuton (12 mg/kg) in serum (**A**) and renal tissue (**B**) following multiple intraperitoneal doses in rats. Data are shown as mean ± SD. *N* = 6 for serum data points and *N* = 2 for renal tissue data points. Steady state = 10th consecutive daily dose.

To assess dose accumulation, zileuton (12 mg/kg) was given once daily with the 1 mg/mL formulation for 10 days. Compared to the first dose, no considerable increase in systemic exposure was observed on the 10th day (Fig. 3).

### Attenuation of vancomycin-associated kidney injury

**Fig 4:**
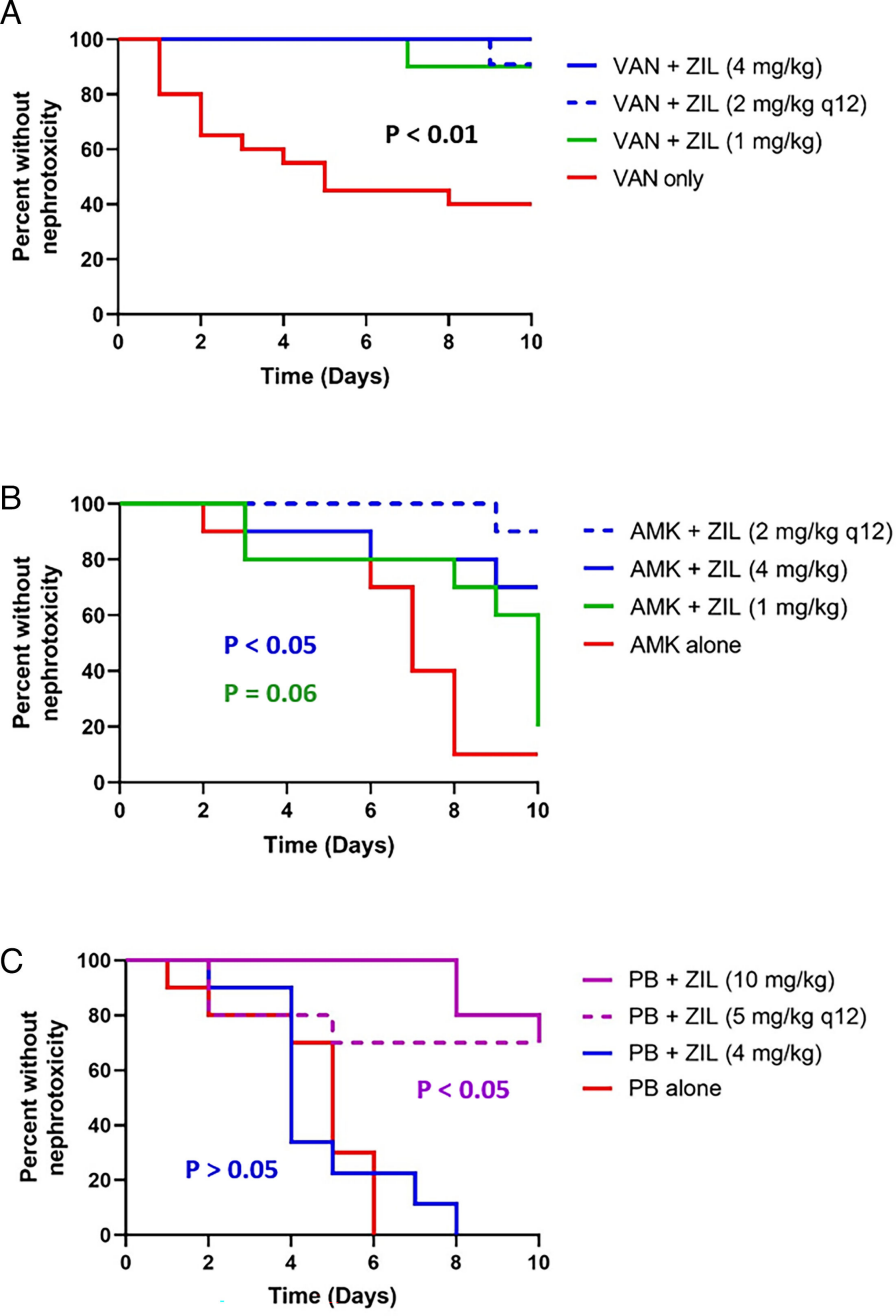
Concomitant zileuton reduces vancomycin- (**A**), amikacin- (**B**), and polymyxin B (**C**)-associated nephrotoxicity in rats. *N* = 20 for VAN alone and *N* = 10 for all other groups. VAN, vancomycin; AMK, amikacin; PB, polymyxin B; ZIL, zileuton. Kaplan-Meier survival analyses compared to antibiotic alone. Zileuton doses were given every 24 hours unless otherwise noted.

When given vancomycin (200 mg/kg/day) alone, 60% of animals developed nephrotoxicity by day 10. Zileuton significantly delayed the onset of nephrotoxicity (*P* < 0.01) (Fig. 4A). The percentage of animals developing nephrotoxicity was reduced from 60% to ≤10%. Additionally, the onset of nephrotoxicity was not significantly different between sexes (*P* > 0.05) (data not shown).

### Attenuation of amikacin-associated kidney injury

Administration of amikacin (300 mg/kg/day) alone resulted in 90% of animals developing nephrotoxicity by day 10. Compared to amikacin alone, the onset of nephrotoxicity was significantly delayed by zileuton in a dose-dependent manner (*P* < 0.05). There was a trend of delaying the onset of nephrotoxicity with 1 mg/kg daily of zileuton (*P* = 0.06) (Fig. 4B). Zileuton reduced the percentage of animals developing nephrotoxicity from 90% to 30% (4 mg/kg every 24 h) and 10% (2 mg/kg every 12 h).

### Attenuation of polymyxin B-associated kidney injury

When given polymyxin B (20 mg/kg/day) alone, 100% of animals developed nephrotoxicity by day 6. The onset of nephrotoxicity was significantly delayed by zileuton (10 mg/kg every 24 hours and 5 mg/kg every 12 hours) (*P* < 0.05). The number of animals developing nephrotoxicity by day 10 was reduced from 100% to 30%. There was no significant difference in nephrotoxicity with zileuton 4 mg/kg every 24 h (*P* > 0.05) (Fig. 4C). Additionally, the onset of nephrotoxicity was not significantly different between sexes (*P* > 0.05) (data not shown).

## DISCUSSION

The dearth of safe, effective antibiotics available for treating resistant bacterial infections is growing, presenting a major threat to human health. Attenuation of nephrotoxicity associated with glycopeptide, aminoglycoside, and polymyxin antibiotics would allow their optimal use to treat MDR infections. This would be a significant accomplishment for combatting the AMR crisis.

Adjuvant antioxidant/anti-inflammatory compounds have shown promise in reducing antibiotic-associated nephrotoxicity in preclinical models (15, 16). Zileuton, an FDA-approved agent, was previously shown to attenuate cisplatin-associated nephrotoxicity and renal ischemic-reperfusion injury in rodents (7, 17). Zileuton inhibits the leukotriene-producing enzyme, 5-lipoxygenase, thereby reducing the inflammatory response (14, 18). An overexuberant inflammatory response following drug-induced nephrotoxicity has been suggested to exacerbate renal injury (19–21). In this way, zileuton may be able to reduce renal injury. Additionally, zileuton has been shown to have antioxidant and anti-apoptotic properties that may contribute to attenuation of antibiotic-associated nephrotoxicity (7, 22, 23).

Zileuton is water-insoluble, which is deemed to be a major hindrance to its translation to the clinic. In previous studies, zileuton was delivered orally or parenterally using dimethyl sulfoxide (DMSO) as a vehicle. Oral drug delivery is not reliable for the critically ill patient population that may benefit from the renal protective effects of zileuton, and DMSO is not a suitable solvent for parenteral use in humans. Therefore, we developed a proprietary cosolvent formulation for zileuton, where it is first dissolved in ethanol and can be stored at −20°C long term. This stock ethanol solution is then diluted with an aqueous cosolvent mixture suitable for parenteral administration shortly prior to dosing. In humans, we envision this zileuton formulation will be delivered by intermittent intravenous infusions. Zileuton was delivered intraperitoneally to rats in this study as a proof of concept, which would also mimic the pharmacokinetic profiles after intermittent intravenous infusions. The zileuton formulations showed good multi-dose tolerability when administered by intraperitoneal injection, and minimal hemolysis (<20%) *in vitro* supporting the safety of intravenous dosing in future studies.

We analyzed the serum and renal tissue pharmacokinetics of zileuton following intraperitoneal administration of zileuton (4 and 12 mg/kg) using the 1 mg/mL formulation. A proportional increase in Cmax and AUC was observed between the doses in rats, suggestive of linear pharmacokinetics. Notably, drug exposure observed in renal tissue was similar to that in the serum. Using the serum AUC, we calculated HEDs. A 600 mg oral dose of zileuton in humans results in an AUC of 19.2 mg*h/L (14). Assuming dose linearity and similar bioavailability (between intraperitoneal administration in rats vs oral administration in humans), 4 and 12 mg/kg intraperitoneal doses in rats correlate to 880 and 3,000 mg oral doses in humans, respectively. The maximum approved daily dose of zileuton in humans is 2400 mg. Therefore, zileuton systemic exposures observed in rats were comparable to those after typical doses in humans.

Based on previous pharmacokinetic studies and our unpublished data, the doses of antibiotics chosen for our studies are comparable to human doses, assuming dose linearity (24, 25). A significant reduction in vancomycin-associated nephrotoxicity was observed at a zileuton dose as low as 1 mg/kg (200 mg in humans). Amikacin required a zileuton dose of 4 mg/kg (900 mg in humans) to delay nephrotoxicity. Reduction in polymyxin B-associated nephrotoxicity required a zileuton dose of 10 mg/kg (2,500 mg in humans). Polymyxin B is significantly more nephrotoxic than amikacin and vancomycin. We reason that this may explain why a higher dose of zileuton is required. Zileuton dose fractionation (i.e., 12 hour dosing instead of 24 hour dosing) generally resulted in similar renal protection overall (*P* > 0.05). This would imply that zileuton renal protection is not predominantly dependent on Cmax and could also allow more flexibility with the dosing schedule in a clinical setting.

Limitations of our study include the use of only one biomarker of kidney injury (i.e., sCr) and only one antibiotic in each class. The inclusion of other biomarkers and/or glomerular filtration rate studies could increase confidence in our findings and are being incorporated into future experiments. As we removed animals from the study once they developed nephrotoxicity, we were not able to perform a quantitative comparison of sCr over time. Additionally, we do not have pharmacokinetic data on the antibiotics when they are co-administered with zileuton. Finally, we do not yet have safety or pharmacokinetic data when zileuton is administered intravenously to support translation to humans and calculate the bioavailability of intraperitoneal delivery. Future directions include histopathological analysis to confirm reduced renal injury, pharmacokinetic analysis when zileuton is co-administered with antibiotics, pharmacokinetic/safety analysis of intravenous zileuton, and ascertaining the mechanism(s) of zileuton renal protection.

### Conclusions

These preliminary development studies support that zileuton has the potential to be used as a parenteral adjuvant to attenuate glycopeptide-, aminoglycoside-, and polymyxin-associated nephrotoxicity. This would allow the optimal use of these antibiotics to treat difficult infections and make a significant contribution to combating the AMR crisis. Further experiments are warranted to repurpose zileuton as a renal protective drug.
